# The clinicopathological characteristic associations of long non-coding RNA gene H19 polymorphisms with uterine cervical cancer

**DOI:** 10.7150/jca.36707

**Published:** 2019-10-15

**Authors:** Ming-Chao Huang, Ying-Hsiang Chou, Huang-Pin Shen, Soo-Cheen Ng, Yueh‐Chun Lee, Yi-Hung Sun, Chun-Fang Hsu, Shun-Fa Yang, Po-Hui Wang

**Affiliations:** 1 Institute of Medicine, Chung Shan Medical University, Taichung, Taiwan; 2 Department of Obstetrics and Gynecology, Hsinchu MacKay Memorial Hospital, Hsinchu, Taiwan; 3 Mackay Medicine, Nursing, and Management College, Taipei, Taiwan; 4 Department of Medical Imaging and Radiological Sciences, Chung Shan Medical University, Taichung, Taiwan; 5 Department of Radiation Oncology, Chung Shan Medical University Hospital, Taichung, Taiwan; 6 Department of Obstetrics and Gynecology, Chung Shan Medical University Hospital, Taichung, Taiwan; 7 School of Medicine, Chung Shan Medical University, Taichung, Taiwan; 8 Department of Obstetrics and Gynecology, Chi-Mei Foundation Medical Center, Tainan, Taiwan; 9 Department of Medical Research, Chung Shan Medical University Hospital, Taichung, Taiwan

**Keywords:** long non-coding RNA gene H19, uterine cervical cancer, clinicopathological characteristics, lymph node metastasis, 5 year survival

## Abstract

The purposes of the current study were conducted to explore the relationships among long non-coding RNA gene H19 (LncRNA H19) polymorphisms and clinicopathological characteristics of uterine cervical cancer, and patient prognosis in Taiwan. Five genetic variants of LncRNA H19 rs3024270, rs2839698, rs3741219, rs2107425 and rs217727 were recruited from one hundred and thirty-four patients with invasive cancer, 101 with high-grade cervical intraepithelial neoplasia (CIN) of uterine cervix and 325 controls and their genetic distributions were determined. It indicated no associations of these LncRNA H19 genetic variants with development of cervical cancer. CC/CT in LncRNA H19 rs2839698 exhibited less risk to have pelvic lymph node metastasis [Odds ratio (OR): 0.19, 95% Confidence interval (CI):0.04-0.82, *p*=0.028)], as compared with TT. Meanwhile, cervical cancer patients with AA/AG in rs3741219 also had less risk to develop pelvic lymph node metastasis (OR: 0.17, 95% CI: 0.05-0.63, *p*=0.008), large tumor (OR: 0.17, 95% CI: 0.04-0.82, *p*=0.014) as well as parametrium (OR: 0.26, 95% CI: 0.07-0.95, *p*=0.045) and vagina invasion (OR: 0.25, 95% CI: 0.07-0.91, *p*=0.041, as compared to those with GG. However, only positive pelvic lymph node metastasis was related to worse recurrence-free survival and poor overall survival. Conclusively, it indicated no association of LncRNA H19 SNPs with cervical carcinogensis in Taiwanese women. Although genotypes TT in LncRNA H19 rs2839698 and GG in rs3741219 are related to some poor clinicopathological parameters of cervical cancer, only pelvic lymph node status could predict 5 year patient survival significantly.

## Introduction

Uterine cervical cancer was regarded as the most common gynecological cancer, if carcinoma in situ (CIS) was recruited, based on 2013 cancer registry annual report in Taiwan. Cervical carcinogenesis is a multistep process of neoplastic transition from cervical intraepithelial neoplasia (CIN), which was considered as precancerous lesions, to invasive cancer, which was found to be the end of this progressive intraepithelial dysplastic atypia 1, 2. The histological diagnoses include CIN1 (low-grade CIN or mild dysplasia; mitoses and immature cells in the lower one-third of cervical epithelium) as well as high-grade CIN including CIN 2 (moderate dysplasia) and CIN 3 (severe dysplasia and CIS), which define involvement of mitoses and immature cells in middle and upper third respectively, were in accordance with their cytological counterparts, low-grade squamous cell intraepithelial lesions (LSIL) and high-grade squamous cell intraepithelial lesions (HSIL) 3.

Long non-coding RNA gene H19 (LncRNA gene H19), a 2.7 kb gene which expresses maternally and imprints paternally, is situated near the telomeric region of chromosome 11p15.5 and encodes a 2.3 kb long, capped, spliced, and polyadenylated noncoding RNA 4, 5. The transcript of this gene LncRNA H19 was found by Brannan and is one of the first discovered LncRNAs 4, 6. It indicated that H19 displays critical roles in embryonic growth and development tissues 7, 8 and is overexpressed in a variety of cancers, including gastric and breast cancers 9, 10. MiR-675, which is known as one of the most important transcripts in the H19 locus, is located at exon 1 and implicated in the carcinogenesis and tumor invasiveness 11. LncRNA H19 has been reported to be predictive of clinicopathological characteristics and H19 expression was associated with histological grades, clinical stages and lymph node metastasis status using a meta-analysis 12, 13.

Single nucleotide polymorphism (SNP) variants mean the form of DNA variation in human 14. When genetic variants present in exon, promoter regions or 3'-untranslated region of a gene, they may exert the impacts on gene expression and biological processes 14. Some SNPs within LncRNA genes have been identified to affect the expression and function of LncRNAs, which are known as regulatory RNAs longer than 200 nucleotides without protein-coding potential, and therefore have an influence on individual cancer susceptibility and patient prognosis 13, 15-17. Recently, some studies demonstrated that LncRAN H19 SNPs was related to cancer development. To our knowledge, few studies investigate the clinical implication of H19 SNPs in uterine cervical cancer in Taiwan. Then, we inferred that H19 SNPs was associated with the development and progression of cervical cancer. We therefore conducted this study to investigate the relationships among H19 SNPs, cervical carcinogenesis and clinicopathological variables and patient prognosis.

## Materials and Methods

### Data source and participants

The individuals, enrolling 134 invasive cancer and 101 high-grade CIN of uterine cervix as well as 325 normal controls, were recruited consecutively from the Department of Obstetrics and Gynecology in Taichung Chung Shan Medical University Hospital, Taiwan from February 1994 to December 2014 in this retrospective study. Invasive cancer patients underwent the standard treatment protocols that were revised from National Comprehensive Cancer Network guidelines.

Cervical punch biopsy under colposcopy was performed for patients suspected to have invasive cancer or precancerous lesions and the diagnoses for these patients were clarified by pathology report. Normal controls were enrolled when they received Papanicolaou smears at outpatient department for general examination in same hospital and the normal cytologic diagnosis was compatible with and confirmed by normal colposcopic findings. Chung Shan Medical University Hospital institutional review board approved this study (CSMUH No: CS18208).

### Selection of H19 genetic variants and DNA extraction from all participants

Five genetic polymorphisms of LncRNA H19 were selected based on the International HapMap Project dbSNP database for the current study. Furthermore, these SNPs of LncRNA H19 gene were selected since these SNPs were associated with the progression of the various cancers 18-21. Genetic variants rs3024270, rs2839698, rs3741219, rs2107425 and rs217727 were included for genotypic determination. Hardy-Weinberg equilibrium value ≥ 0.05 was necessary.

Standard venipuncture technique was performed by the staffs to collect blood samples from all subjects and they were deposed into Vacutainer tubes merged with ethylenediaminetetraacetic acid. The specimens were immediately stored at 4℃. DNA was extracted from leukocytes based on previous protocols 22. After extraction, DNA was dissolved into pH 7.8 TE buffer. Thereafter, it was quantified by the measurement of OD260. The OD260/OD280 ratio was then determined and the range of 1.8-2.0 met our criteria and thought to be pure to prevent its cross reactivity from the present homologous RNA in the specimens. The final products were then stored at -20°C and were applied as templates for the polymerase chain reaction (PCR).

### Statistical analysis

Analysis of variance (ANOVA) was performed to compare the age distribution of the studied subjects with Tukey test for post hoc analysis. Hardy-Weinberg equilibrium was applied to detect the genotype frequencies of rs3024270, rs2839698, rs3741219, rs2107425 and rs217727 in normal controls [degree of freedom (d.f.)=2]. Chi-square or Fisher exact tests were used to investigate the associations between various H19 SNPs frequencies and the development of cervical neoplasia (comprising including invasive cancer and precancerous lesions) or among H19 distributions, cervical invasive cancer and precancerous lesions and normal controls. These tests were also used to associate H19 SNPs distributions with various clinicopathological characteristics.

A Cox proportional hazard model was used to assess the effects of H19 SNPs on the recurrence-free survival or overall survival after adjusting various clinicopathological characteristics in relation to follow-up intervals. In addition, Kaplan-Meier curves were used to plot the effect of H19 SNPs and significant clinicopathological variables for 5 year survival. The log-rank test was used to detect the differences between these curves. Hazard ratios (HRs) and their 95% confidence intervals (CI) were examined by the SPSS, version 12.0. A significant difference was defined if *p* value <0.05.

## Results

### Age distribution and Hardy-Weinberg equilibrium

Patients with cervical neoplasias exhibited significantly different age distribution from normal control women (50.3 ± 13.6 vs. 44.2 ± 10.4, *p*<0.001). It indicated that the age distribution was statistically different between patients with cervical cancer and control women (55.0 ± 12.2 vs. 44.2 ± 10.4, *p*<0.001) as well as between patients with cervical invasive cancer and those with precancerous lesions (55.0 ± 12.2 vs. 44.1 ± 12.9, *p*<0.001), but not statistically different between those with precancerous lesions and control women (44.1 ± 12.9 vs. 44.2 ± 10.4, *p*= 0.995), applied by the ANOVA using Tukey test for post hoc analysis.

The minor allele frequencies of H19 genetic polymorphisms rs3024270, rs2839698, rs3741219, rs2107425 and rs217727 in control women were all ≥5 %. In these controls group, genotypic frequency of H19 SNP rs3024270 satisfy the Hardy-Weinberg equilibrium [χ^2^ value, 1.020,* p*= 0.601; d.f.=2]. The distributions of H19 SNPs rs2839698, rs3741219, rs2107425 and rs217727 were also conform to the Hardy-Weinberg equilibrium (χ^2^ value, 0.015,***p***= 0.993; χ^2^ value, 0.361, ***p***= 0.835; χ^2^ value, 0.450, ***p***= 0.799 and χ^2^ value, 0.154, ***p***= 0.926, respectively).

### Association of H19 genetic polymorphisms with development of cervical cancer

Table 1 shows the genotypic distributions of LncRNA H19 SNPs in the Taiwanese women with cervical neoplasias and the controls. No significantly different frequencies of rs3024270, rs2839698, rs3741219, rs2107425 and rs217727 were exhibited between patients with cervical neoplasias and normal controls. Even after controlling for the age, no significant differences were still present for these SNPs between patients with cervical neoplasias and controls.

Although the cervical neoplasia group was subdivided into invasive cancer and precancerous lesion subgroups, no significant differences existed for the distributions of H19 rs3024270, rs2839698, rs3741219, rs2107425 and rs217727 among patients with invasive cancer and precancerous lesions as well as normal controls (Table 2). Even controlling for age, no significant differences were present for these SNPs.

### Relationships of LncRNA H19 with clinicopathological characteristics

Patients with cervical invasive cancer, who present with CC/CT in LncRNA H19 rs2839698, had less risk to develop pelvic lymph node metastasis, as compared to those with TT (OR: 0.19, 95% CI: 0.04-0.82, *p*=0.028; Table 3). Meanwhile, cervical cancer patients with AA/AG in rs3741219 also exhibited less risk to develop pelvic lymph node metastasis, as compared to those with GG (OR: 0.17, 95% CI: 0.05-0.63, *p*=0.008; Table 3). In addition, these patients exerted less risk to have large tumor (OR: 0.17, 95% CI: 0.04-0.82, *p*=0.014), parametrium invasion (OR: 0.26, 95% CI: 0.07-0.95, *p*=0.045) and vagina invasion (OR: 0.25, 95% CI: 0.07-0.91, *p*=0.041; Table 3). However, there were no significant associations of the distribution of LncRNA H19 rs3024270, rs2107425 and rs217727 with clinicopathological characteristics.

### Impact of H19 genetic polymorphisms and recurrence-free survival and overall survival of the patients with cervical invasive cancer

In correlating LncRNA H19 rs2839698 and rs3741219 with patient prognosis, Cox proportional hazard model was applied. However, rs2839698 and rs3741219 were not associated with recurrence-free survival of cervical cancer patients (*p*=0.932 and 0.935 respectively; Table 4). H19 rs2839698 and rs3741219 also could not predict patient overall survival (*p*=0.961 and 0.959 respectively). Only positive pelvic lymph node metastasis was related to worse recurrence-free survival (HR: 2.81, 95% CI: 1.09-7.26, *p*=0.033) and poor overall survival (HR: 4.79, 95% CI: 1.81-12.66, *p*=0.002; Table 4).

**Figure 1:**
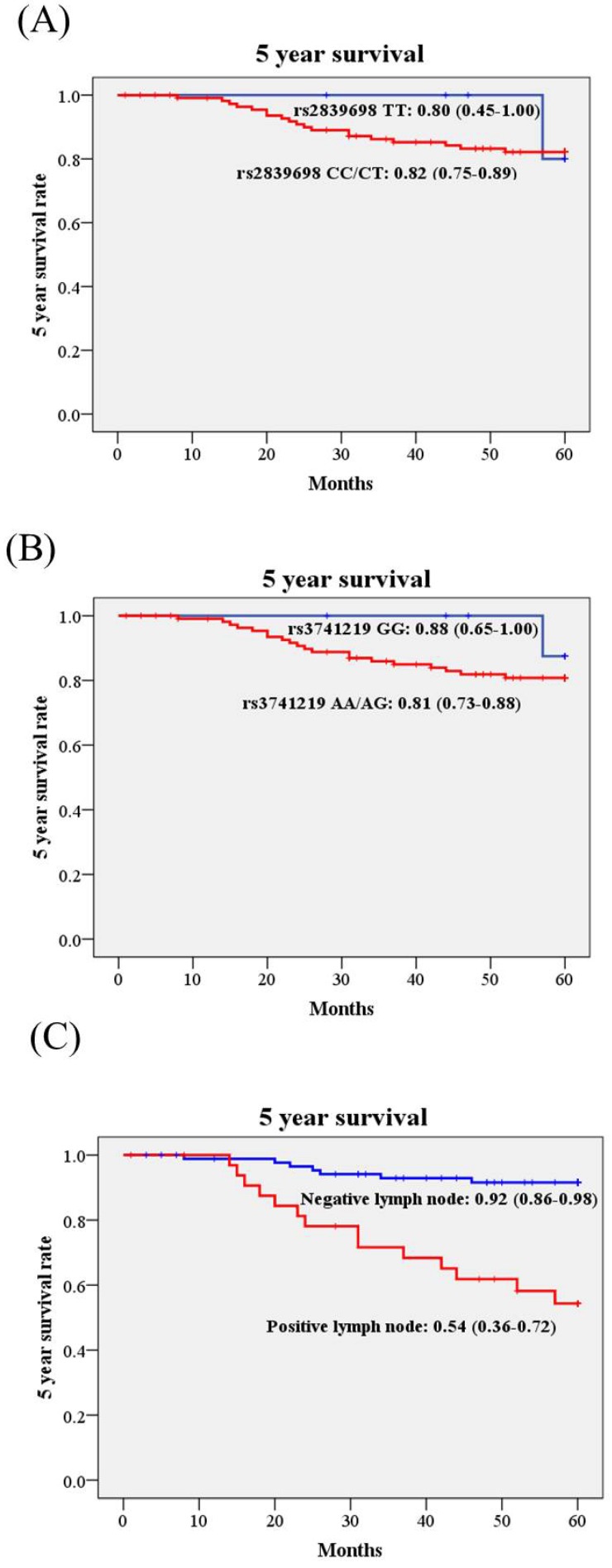
** Kaplan-Meier curves for 5 year survival rate based on long non-coding RNA gene H19 polymorphisms and pelvic lymph node status.** (A), rs2839698 CC/CT vs. TT, 5 year survival rate: 0.82, 95% CI: 0.75-0.89 vs. 0.80, 95% CI: 0.45-1.00; *p*=0.722); (B), rs3741219 AA/AG vs. GG, 5 year survival rate: 0.81, 95% CI: 0.73-0.88 vs. 0.88, 95% CI: 0.65-1.00; *p*=0.429); (C), pelvic lymph node status, positive vs. negative, 5 year survival rate: 0.54, 95% CI: 0.36-0.72 vs. 0.92, 95% CI: 0.86-0.98; *p*<0.001). Log-rank test was applied to detect statistical significance. 95% CI, 95% confidence interval.

Kaplan-Meier curves were used to plot the influence of H19 SNPs and clinicopathological variables on 5 year survival rate in cervical cancer patients. Although H19 rs3741219 were associated with some clinicopathological parameters, the 5 year survival rates were not different in patients with different genotypes. The 5 year survival rate of patients with AA/AG was 0.81 (95% CI: 0.73-0.88), while those with GG 0.88 (95%: 0.65-1.00; Figure 1). Similar effect was noted in rs2839698. Only patients with different lymph node status present different 5 year survival rate significantly (Figure 1C). The 5 year survival rate of cervical cancer patients with positive lymph node metastasis was only 0.54 (95% CI: 0.36-0.72) as compared to those with negative lymph node metastasis 0.92 (95% CI: 0.86-0.98; Figure 1C).

## Discussion

It indicated that there were no significantly different frequencies of LncRNA H19 SNPs rs3024270, rs2839698, rs3741219, rs2107425 and rs217727 between patients with cervical neoplasias and controls in Taiwan in the current study. Even after the cervical neoplasia group was classified into invasive cancer and precancerous lesion subgroups, it still displayed no significant differences for the genotypic frequencies of these H19 SNPs among patients with invasive cancer and precancerous lesions as well as normal controls. They were not involved in cervical carcinogenesis. Using meta-analysis, Lu et al. found that H19 SNP rs217727 was not significantly correlated with cancer risk in dominant (TT + CT vs. CC) and recessive (TT vs. CT + CC) genetic models 23. However, genotypes CT/TT have been reported to be correlated with reduced risk of breast cancer 24 and elevated risk of gastric and bladder cancers 25, 26. Guo et al. found that H19 SNP rs217727 is significantly associated with the susceptibility of oral squamous cell carcinoma in Chinese population 27. In contrast to our findings, Jin et al. demonstrated that the genotypes CT/TT in H19 SNP rs217727 carried the elevated risk of cervical cancer (OR: 1.62, 95% CI: 1.15-2.29; *p*=0.0059) in Chinese population as compared to CC 28. H19 rs217727 is located on the exon 5 29. SNPs in the exon or promoter area of H19 gene may influence the expression of H19 or the structure and function of the H19 RNA 19, 21, 29. This is supported by the cumulative evidence to reveal that abnormal H19 expression is found in bladder cancer and hepatocellular carcinoma, and increases cancer cell proliferation, indicating an oncogenic function 7, 30, 31. H19 SNP rs2839698 mutation has been reported to reduce the risk of bladder cancer 32. However, Li et al. demonstrated that H19 SNP rs2839698 was related to increased risk of colorectal cancer in Chinese population 29. Moreover, H19 rs210742 was found to be associated with the risk of lung cancer significantly 33. But, H19 SNP rs3741219 was not found to be associated with the risk of breast cancer 24.

H19 SNP rs2839698 is located within the 3ʹ untranslated region (3ʹ UTR) of exon 1 of LncRNA gene H19 and probably affects the function of H19 gene to some extents through the changes of crucial folding structures and targeting miRNA 29. Variants in 3ʹ UTR of H19 SNP rs2839698 leads to the loss of hsa-miR-24-1-5p and hsa-miR-24-2-5p function, which present as tumor suppressors and therefore their reduced expressions may promote invasiveness in some cancers such as colorectal and bladder cancers 34, 35. Xia et al. revealed that the base A mutated to G in H19 SNP rs3741219, which is located within exon 5, may create hsa-miR-1539 miRNA binding sites on H19 and probably changes the LncRNA structure, influences its stability and affects miRNA-LncRNA interaction 24. The functions of H19 can be dissected into two main actions; in addition to presenting as a modulator of micro-RNAs or proteins via their binding, H19 is also the mainly precursor of miR-675 and may determine its level, which possesses multiple targets in diverse signaling pathways, and then regulates a variety of biological processes via target inhibition by miR-675 36, 37. Reduced miR-675 expression has been reported to be correlated with invasion of hepatocellular carcinoma 38. Furthermore, H19 may be involved in epithelial to mesenchymal transition and promotes invasion and metastasis of some solids cancers such as pancreatic cancer and hepatocellular carcinoma 38-43.

The elevated expression of LncRNA H19 was reported to be associated with poor prognosis in gastric cancer, cervical cancer and lung cancer 9, 44, 45. Our study revealed that LncRNA H19 SNPs rs2839698 and rs3741219 were related to pelvic lymph node metastasis, the most critical prognostic parameter for the survival of cervical cancer patients 46, 47. However, they could not predict patient survival significantly in the present study. Yang et al. demonstrated no significant association of H19 SNPs with overall survival of patients with hepatocellular carcinoma 48. Until now, no study presents a significant association of H19 SNPs with survival of cervical cancer patients.

There were some limitations in the current research. Firstly, we only studied the central Taiwan population and did not recruit residents of other areas. Secondly, the sample size was relatively small especially for the invasive cancer and precancerous groups. This may restrict the possible subgroup analysis. Thirdly, because the controls were enrolled from outpatient department for general examination in our hospital and there was no information of the human papillomavirus infection, which could not be analyzed for the impact of this factor.

In conclusion, the present research reveals no association of LncRNA H19 SNPs with cervical carcinogensis in Taiwanese women. However, genotypes TT in LncRNA H19 rs2839698 and GG in rs3741219 are related to some poor clinicopathological parameters of cervical cancer. But they cannot predict patient survival significantly.

## Figures and Tables

**Table 1:** Genetic variant frequencies of the H19 gene in Taiwanese women with uterine cervical neoplasias and normal controls

Genetic polymorphisms	Normal controls (n =325)	Cervical neoplasias^a^ (n=235)	ORs (95% CIs)	*p* values	AORs (95% CIs)^b^	Adjusted *p* values
**rs3024270**				0.512		0.371
CC^c^	71	51	1.00		1.00	
CG	150	120	1.11 (0.72-1.72)		1.11 (0.71-1.74)	0.651
GG	95	60	0.88 (0.54-1.43)		0.82 (0.50-1.36)	0.445
CC^c^	71	51	1.00	0.914	1.00	0.984
CG/GG	245	180	1.02 (0.68-1.54)		1.00 (0.65-1.52)	
GG^c^	95	60	1.00	0.295	1.00	0.183
CC/CG	221	171	1.23 (0.84-1.79)		1.31 (0.88-1.94)	
**rs2839698**				0.936		0.981
CC^c^	154	115	1.00		1.00	
CT	134	99	0.99 (0.69-1.41)		1.04 (0.72-1.50)	0.849
TT	30	20	089 (0.48-1.65)		1.00 (0.72-1.50)	0.991
CC^c^	154	115	1.00	0.868	1.00	0.866
CT/TT	164	119	0.97 (0.69-1.36)		1.03 (0.73-1.46)	
TT^c^	30	20	1.00	0.720	1.00	0.967
CC/CT	288	214	1.12 (0.62-2.02)		1.01 (0.55-1.87)	
**rs3741219**				0.875		0.895
AA^c^	152	112	1.00		1.00	
AG	130	100	1.04 (0.73-1.49)		1.09 (0.75-1.58)	0.649
GG	32	21	0.89 (0.49-1.63)		1.00 (0.54-1.88)	0.989
AA^c^	152	112	1.00	0.937	1.00	0.692
AG/GG	162	121	1.01 (0.72-1.42)		1.07 (0.76-1.53)	
GG^c^	32	21	1.00	0.645	1.00	0.906
AA/AG	282	212	1.15 (0.64-2.04)		1.04 (0.57-1.89)	
**rs2107425**				0.684		0.474
CC^c^	109	88	1.00		1.00	
CT	155	107	0.86 (0.59-1.24)		0.79 (0.54-1.17)	0.236
TT	48	38	0.98 (0.59-1.63)		0.94 (0.56-1.59)	0.823
CC^c^	109	88	1.00	0.496	1.00	0.308
CT/TT	203	145	0.89 (0.62-1.26)		0.83 (0.57-1.19)	
TT^c^	48	38	1.00	0.770	1.00	0.762
CC/CT	264	195	0.93 (0.59-1.48)		0.93 (0.58-1.50)	
**rs217727**				0.875		0.855
CC^c^	135	102	1.00		1.00	0.632
CT	139	103	0.98 (0.68 (1.41)		0.91 (0.63-1.33)	0.663
TT	39	28	0.95 (0.55-1.65)		0.88 (0.50-1.56)	
CC^c^	135	102	1.00	0.880	1.00	0.584
CT/TT	178	131	0.97 (0.69-1.37)		0.91 (0.63-1.29)	
TT^c^	39	28	1.00	0.876	1.00	0.771
CC/CT	274	205	1.04 (0.62-1.75)		1.08 (0.63-1.85)	

Statistical analysis: logistic regression model or chi-square test. ^a^Cervical neoplasias included precancerous lesions and invasive cancer of the uterine cervix. ^b^The adjusted *p* values as well as adjusted odds ratios and their 95% confident intervals were examined by logistic regression model after controlling for age. ^c^Used as a reference for comparison to determine the odds ratios of other genotypes. 95% CIs, 95% confidence intervals.

**Table 2:** Genetic variant frequencies of H19 in Taiwanese women with invasive cancer or precancerous lesions of uterine cervix and normal controls

Genetic polymorphisms	Normal controls (n =325)	Pre-cancerous lesions (n =101)	Invasive cancer (n =134)	*p* values	AORs (95% CIs)^a^	Ad. *p* values	AORs (95% CIs)^b^	Ad. *p* values
**rs3024270**								
CC^c^	71	25	26	0.629	1.00		1.00	
CG	150	51	69		1.41 (0.81-2.45)	0.228	1.31 (0.78-2.20)	0.299
GG	95	23	37		1.46 (0.77-2.78)	0.252	0.98 (0.51-1.86)	0.945
CC^c^	71	25	26	0.601	1.00		1.00	
CG/GG	245	74	106		0.86 (0.51-1.45)	0.565	1.22 (0.70-2.11)	0.490
GG^c^	95	23	37	0.419	1.00		1.00	
CC/CG	221	76	95		1.42 (0.84-2.41)	0.188	1.20 (0.74-1.96)	0.455
**rs2839698**								
CC^c^	154	46	69	0.820	1.00		1.00	
CT	134	45	54		1.01 (0.46-2.22)	0.988	1.11 (0.47-2.60)	0.815
TT	30	10	10		0.89 (0.41-1.97)	0.781	1.15 (0.50-2.67)	0.739
CC^c^	154	46	69	0.622	1.00		1.00	
CT/TT	164	55	64		1.12 (0.72-1.76)	0.611	0.94 (0.61-1.47)	0.798
TT^c^	30	10	10	0.769	1.00		1.00	
CC/CT	288	91	123		0.95 (0.45-2.01)	0.886	1.13 (0.50-2.56)	0.764
**rs3741219**								
AA^c^	152	44	68	0.869	1.00		1.00	
AG	130	46	54		1.22 (0.76-1.97)	0.407	0.98 (0.62-1.56)	0.928
GG	32	9	12		0.97 (0.43-2.20)	0.947	1.01 (0.46-2.22)	0.988
AA^c^	152	44	68	0.634	1.00		1.00	
AG/GG	162	55	66		1.17 (0.75-1.85)	0.489	0.98 (0.63-1.53)	0.939
GG^c^	32	9	12	0.899	1.00		1.00	
AA/AG	282	90	122		1.13 (0.52-2.47)	0.752	0.99 (0.46-2.12)	0.969
**rs2107425**								
CC^c^	109	39	49	0.751	1.00		1.00	
CT	155	43	64		0.77 (0.47-1.27)	0.310	0.83 (0.51-1.36)	0.461
TT	48	19	19		1.10 (0.58-2.10)	0.770	0.85 (0.43-1.67)	0.633
CC^c^	109	39	49	0.771	1.00		1.00	
CT/TT	203	62	83		0.85 (0.53-1.35)	0.492	0.84 (0.53-1.33)	0.449
TT^c^	48	19	19	0.629	1.00		1.00	
CC/CT	264	82	113		0.79 (0.44-1.41)	0.421	1.06 (0.57-1.96)	0.853
**rs217727**								
CC^c^	135	45	57	0.900	1.00		1.00	
CT	139	41	62		0.88 (0.54-1.44)	0.617	0.96 (0.60-1.54)	0.875
TT	39	14	14		1.07 (0.53-1.26)	0.842	0.75 (0.36-1.57)	0.447
CC^c^	135	45	57	0.937	1.00			
CT/TT	178	55	76		0.93 (0.59-1.46)	0.736	0.91 (0.58-1.43)	0.695
TT^c^	39	14	14	0.739	1.00		1.00	
CC/CT	274	86	119		0.88 (0.45-1.69)	0.692	1.30 (0.65-2.61)	0.452

^a^Adjusted *p* values and adjusted odds ratios with their 95% CIs were examined using multiple and multinomial logistic regression models after controlling for age between patients with uterine cervical precancerous lesions and control women. ^b^Adjusted *p* values and adjusted odds ratios with their 95% CIs were examined using multiple and multinomial logistic regression models after controlling for age between patients with uterine cervical invasive cancer and control women. ^c^Used as a reference for comparison to determine the odds ratios of other genotypes. AORs, adjusted odds ratios; 95% CIs, 95% confidence intervals; Ad. *p*, adjusted *p*.

**Table 3:** Relationships of genotypic distribution of H19 rs2839698 and rs3741219 with clinicopathological characteristics of the patients with uterine cervical invasive cancer

Characteristics^a^	rs2839698		ORs (95% CIs)	*p* value	rs3741219			ORs (95% CIs)	*p* value
	TT^b^	CC/CT			GG^b^	AA/AG			
**Clinical stage**				0.122					0.091
stage I^b^	2	73	1.00		3	72	1.00		
≥ stage II	5	47	0.26 (0.05-1.38)		7	46	0.27 (0.07-1.11)		
**Pathologic type**				0.634					1.00
squamous cell carcinoma^b^	6	99	1.00		9	97	1.00		
adenocarcinoma	2	21	0.64 (0.12-3.37)		2	21	0.97 (0.20-4.84)		
**Cell grading**				0.617					1.00
well (grade 1)^b^	2	19	1.00		2	19	1.00		
moderate & poor (grades 2/3)	6	101	1.77 (0.33-9.45)		9	99	1.16 (0.23-5.79)		
**Stromal invasion depth**				0.724					0.591
≤10 mm^b^	3	55	1.00		6	53	1.00		
>10 mm	5	62	0.68 (0.15-2.96)		5	62	1.40 (0.41-4.86)		
**Tumor diameter^b^**				0.142					0.014^c^
≤ 4cm	2	67	1.00		2	67	1.00		
>4cm	6	53	0.26 (0.05-1.36)		9	51	0.17 (0.04-082)		
**Parametrium**				0.117					0.045^c^
no invasion^b^	3	80	1.00		4	79	1.00		
invasion	5	37	0.28 (0.06-1.22)		7	36	0.26 (0.07-0.95)		
**Vagina**				0.117					0.041^c^
no invasion^b^	3	82	1.00		4	82	1.00		
invasion	5	38	0.28 (0.06-1.22)		7	36	0.25 (0.07-0.91)		
**Pelvic lymph node**				0.028^c^					0.008^c^
no metastasis^b^	3	91	1.00		4	90	1.00		
metastasis	5	28	0.19 (0.04-0.82)		7	27	0.17 (0.05-0.63)		
									

Statistical analyses: chi-square or Fisher's exact tests, ^c^*p*<0.05. ^a^Some clinicopathological data could not be obtained from the patients with cervical invasive cancer due to incomplete medical charts or records. ^b^As a reference. ORs, odds ratios; 95% CIs, 95% confidence intervals.

**Table 4:** Analysis for the associations of H19 genetic variants rs2839698 and rs3741219 as well as various clinicopatholgical characteristics with the recurrence-free survival and overall survival of the patients with uterine cervical cancer

	Recurrence-free survival			Overall survival	
Variables	*p* value	HR & 95% CI^b^		*p* value	HR & 95% CI^b^
**H19 genetic polymorphisms**	0.932	-		0.961	-
rs2839698 CC/CT vs. TT^a^					
rs3741219 AA/AG vs. GG^a^	0.935	-		0.959	-
**Clinicopathological characteristics**					
**Pelvic lymph node**					
metastasis vs. no metastasis^a^	0.033	2.81 (1.09-7.26)		0.002	4.79 (1.81-12.66)

Statistical analyses: Cox proportional hazard model. ^a^As a comparison reference. ^b^HR, hazard ratio and 95% CI, 95% confidence interval for H19 genetic variants rs2839698 and rs3741219 and clinicopathological variables, compared to their respective controls.
